# Differential Inhibition of the TGF-β Signaling Pathway in HCC Cells Using the Small Molecule Inhibitor LY2157299 and the D10 Monoclonal Antibody against TGF-β Receptor Type II

**DOI:** 10.1371/journal.pone.0067109

**Published:** 2013-06-27

**Authors:** Francesco Dituri, Antonio Mazzocca, Fernando Juan Peidrò, Patrizia Papappicco, Isabel Fabregat, Flavia De Santis, Angelo Paradiso, Carlo Sabbà, Gianluigi Giannelli

**Affiliations:** 1 Department of Emergency and Organ Transplantation, Section of Internal Medicine Allergology and Immunology, University of Bari Medical School, Bari, Italy; 2 Bellvitge Biomedical Research Institute (IDIBELL), L'Hospitalet, Barcelona, Spain; 3 Clinical Experimental Oncology Laboratory, National Cancer Institute, Bari, Italy; 4 Department Interdisciplinary of Medicine, University of Bari Medical School, Bari, Italy; University of Medicine and Dentistry of New Jersey, United States of America

## Abstract

We investigated blocking the TGF-β signaling pathway in HCC using two small molecule inhibitors (LY2157299, LY2109761) and a neutralizing humanized antibody (D10) against TGF-βRII. LY2157299 and LY2109761 inhibited HCC cell migration on Laminin-5, Fibronectin, Vitronectin, Fibrinogen and Collagen-I and de novo phosphorylation of pSMAD2. LY2157299 inhibited HCC migration and cell growth independently of the expression levels of TGF-βRII. In contrast to LY2157299, D10 showed a reduction in pSMAD2 only after a short exposure. This study supports the use of LY2157299 in clinical trials, and presents new insights into TGF-β receptor cycling in cancer cells.

## Introduction

Hepatocellular carcinoma (HCC) is a lethal cancer, being the third cause of cancer-related death [1]. As a result of improved early detection and screening, the overall survival for this cancer has modestly improved. Nevertheless, the prognosis of patients with advanced disease remains unsatisfactory [2]. Sorafenib is the only approved agent to improve the overall survival of patients with advanced disease [3]. However, the associated side effects of sorafenib, and the rapid progression of disease despite sorafenib treatment, highlight the need for new, additional treatments [4].

Transforming growth factor-beta (TGF-β) signaling occurs following the binding of the TGF-β ligand to TGF-β receptor (R)I that heterodimerizes with the TGF-β RII. This heterodimer complex phosphorylates the intracellular protein Smad-2 and 3, activating a downstream cascade that produces a nuclear transduction protein [5].

TGF-β is an important pathophysiological pathway in the liver associated with fibrogenesis, and promoting extracellular matrix deposition in hepatic stellate cells after viral or metabolic injury. The final outcome of this process is a decreased liver function, which often presents clinically as liver cirrhosis. This loss of liver function commonly precedes the onset of Hepatocellular Carcinoma (HCC) in Western countries [6], [7]. One of the ligands of the TGF-β signaling cascade, TGF-β1, is often detected in blood and urine of patients with HCC and its presence is associated with poor prognosis [8]–[10]. Thus, targeting TGF-β signaling in HCC has been proposed as a novel approach to delay the progression of HCC, and to target the underlying disease which predisposes to HCC [10].

However, a reduced expression of TGF-βRII on the HCC cell surface has been described to be associated with a more aggressive phenotype, while there is still a poor understanding of the role of TGF-β signaling in such a context [11]. Recently, the TGF-βRI kinase inhibitor LY2109761 was found to up-regulate the expression of E-cadherin in HCC cell migration/invasion and the epithelial mesenchymal transition (EMT) in vitro and in vivo models [12]. Furthermore, LY2109761 also blocked the invasion of HCC cells into blood vessels via dephosphorylation of the intracellular tail of β1-integrin at the T788-789 residue [13]. Because the invasion of HCC blood vessels is an important negative prognostic factor, this observation justifies the blocking of TGF-β signaling in HCC [14]–[16]. In fact, LY2109761 demonstrated a stronger anti-angiogenic effect than bevacizumab, resulting in inhibition of tumoral growth [17]. TGF-β signaling also regulates the expression of connective tissue growth factor (CTGF), which is associated with a desmoplastic reaction of the tumor and/or the surrounding tissue microenvironment [18], [19]. In such conditions, LY2109761 inhibits CTGF production, reducing the stromal component of the tumor and slowing the HCC growth *in vivo* [20]. Based on these observations, a phase II clinical trial was initiated in patients with HCC using LY2157299, a second small molecule inhibitor of the TGF-β receptor Type I kinase.

Aim of this study is to characterize two small molecule inhibitors of TGF-β receptor Type I kinase (TGF-βRI) and to compare their activity to a specific TGF-β receptor Type II inhibitor, the monoclonal antibody D10 [21]. Such a strategy of comparing specific inhibitors in HCC has not been previously investigated and may help to better understand the relevance of this pathway in HCC patients. Finally, it may also provide some insight into future use of such inhibitors in HCC.

## Materials and Methods

### Cell Culture and Reagents

HepG2 and HLE human cell lines were obtained from the European Collection of Cell Cultures (ECACC). This cell bank performed cell line certification and cells were propagated in the laboratory for fewer passages before the experiments. Cell cultures were grown in DMEM High Glucose (4,5 g/l) supplemented with L-Glutamine and 10% fetal bovine serum in a humidified atmosphere of 37°C, 5% CO_2_. LY2157299 was kindly provided by Lilly (Indianapolis, IN, USA) and D10, a fully human monoclonal antibody directed against the TGF-β receptor II, was obtained from ImClone Systems (New York, NY, USA). Human recombinant TGF-β1 was purchased from Peprotech (Rocky Hill, NJ, USA). Rabbit polyclonal anti-Phospho-SMAD2, rabbit polyclonal anti-total SMAD2, mouse monoclonal anti-Phospho p44/42, rabbit polyclonal anti p44/p42, rabbit polyclonal anti-Phospho-Akt, and rabbit polyclonal anti-total Akt antibodies were purchased from Cell Signalling technology, Inc. (Beverly, MA, USA). Mouse monoclonal anti-human FAK (pY397) antibody was purchased from BD Transduction Laboratories (Franklin Lakes, NJ USA). Fluorescein isothiocyanate labeled phalloidin was purchased from Sigma Aldrich (St. Louis, MO, USA). Monoclonal antibody directed against human E-Cadherin was obtained from Alexis Biochemicals (San Diego, CA, USA). A polyclonal antibody against p-Smad2 was purchased from Cell Signaling (Denvers, MA). An anti-isotype IgG human was purchased from GenTex (Irvine, CA).

### Analysis of Gene Expression

Total RNA was isolated using the RNeasy Mini Kit (Qiagen,) and underwent DNAse treatment (TURBO DNA-free™ Ambion®). Reverse Transcription (RT) was then carried out with random primers using 1 µg of total RNA from each sample. Quantitative RT-PCR was performed using pre-designed primers and SYBR Green® master mix (Biorad). Housekeeping Glyceraldehyde 3-phosphate dehydrogenase gene (GAPDH) was used as endogenous control. The following primers were obtained from Integrated DNA Technologies® (Leuven, Belgium): **TGFB1**: Forward: 5′- CCG AGA AGC GGT ACC TGA AC-3′. Reverse: 5′- GAG GTA TCG CCA GGA ATT GTT G-3′. **TGFBR2**: Forward: 5′- GCT GAT CAC CGC CTT CCA -3′. Reverse: 5′- CAG GTC CTC CCA GCT GAT GA-3′ **VEGF-A**: Forward: 5′- CAG ATG TCC CGG CGA AGA -3′. Reverse: 5′- GAG GGC GAG TCC CAG GAA-3′. **CTGF:** Forward: 5′- GCA TCC GTA CTC CCA AAA TCT C-3′. Reverse: 5′-GGC AGG GTG GTG GTT CTG T3′. **MMP-2:** Forward: 5′- TGA CCC CAC TGC GGT TTT -3′. Reverse: 5′- GCG GCC AAA GTT GAT CAT G -3′. **GAPDH**: Forward: 5′- CCA CAT CGC TCA GAC ACC AT-3′. Reverse: 5′- GCG CCC AAT ACG ACC AAA T -3′.

### Cell Transfection

HLE and HepG2 cells were transfected with siRNAs targeting the SMAD2, SMAD3 gene or a nonsilencing control (Integrated DNA Technologies) using TransIT-TKO (Mirus, U.S.A.). Eight hours after transfection, cells were serum starved and stimulated with 5 ng/ml of TGF-β1. After forty-eight hours, cells were trypsinized, counted and allowed to migrate on collagen I or Matrigel.

### Cell Migration Assay

Trans-wells migration assays were performed as previously reported [12]. Briefly, trans-well membranes (Corning, U.S.A.) were pre-coated with 10 µg/ml of Collagen I or Matrigel. SMAD2- silenced cells or cells treated with LY2109761, LY2157299 (10 µM) or D10 (25 µg/ml) were incubated in the presence or absence of TGF-β1 (5 ng/ml) for 48 hours before being trypsinized and loaded on the top chamber of the trans-well plates, and allowed to migrate for 16 hours in the presence or absence of a new addition of LY2109761, LY2157299, D10 or TGF-β1. After fixation with 4% paraformaldehyde, migrated cells were stained with crystal violet and quantified.

### Proliferation and Apoptosis Assays

Proliferation assays were performed as previously described [22]. Briefly, 1×10^4^ cells were plated onto 24-well plates. Every two days LY2109761, LY2157299 (10 µM) or D10 (25 µg/ml) were added. Cells were fixed with 4% paraformaldehyde, stained with crystal violet and absorbance was quantified with a spectrophotometer. For apoptosis, cells were treated with LY2109761, LY2157299 (10 µM) or D10 (25 µg/ml) for 48 hours and the TUNEL Detection kit was used according to the manufacturer's instructions (TUNEL Detection kit Genscript, Piscataway, NJ).

### Western Blot Analysis

Total proteins were extracted using a lysis buffer containing 30 mmol/L Tris-HCl (pH 7.5), 5 mmol/L EDTA, 150 mmol/L NaCl, 1% Triton X-100, 0.5% sodium deoxycholate, 0.1% SDS, and 10% glycerol on ice for 30 min. Lysates were then centrifuged at 13,000 rpm for 10 min at 4°C and supernatants were collected. Proteins were quantified by the Bradford protein assay (Biorad), separated in 10% SDS-PAGE and transferred to PVDF membranes. After blocking with 5% w/v nonfat dry milk 1X TBS, 0.1% Tween-20, the membranes were incubated overnight at 4°C with the primary antibody, washed and then incubated with a horse peroxidase-conjugated secondary antibody. The protein bands were visualized using the ECL protein detection (Amersham).

### Ethic Statement

Approval for the study was obtained from the local Ethics Committee of the Azienda Ospedaliero-Universitaria Policlinico, Bari (Apulia) Italy, and patients gave prior written informed consent to the use of their tissues. This study was performed in accordance with the Helsinki Declaration and informed written consent was obtained from all patients. Finally, the data were analyzed anonymously.

### HCC Patients and Tissue Preparations

30 HCC patients, of ages ranging from 59 to 76 years, 12 females and 18 males, were studied. In 26 patients the etiology was viral (15 patients with hepatitis C and 11 patients with hepatitis B), and in four patients alcoholic. Surgical samples were obtained. Tissues were cut in two pieces; one was processed for routine histology, and the other was immediately snap-frozen in liquid nitrogen. Approval for the study was obtained from the local Ethics Committee, and patients gave prior written informed consent to the use of their tissues.

### Immunofluorescence

Expression of type II TGF-β receptor was detected in HCC tissues using the fully human monoclonal antibody D10 (Imclone). Briefly, the frozen tissue sections (8-µm thick) were fixed with PBS containing 4% Paraformaldehyde. Tissues were then stained with primary antibody at 37°C for 30 min., washed 3 times with PBS and incubated with an Alexa Fluor® 594 secondary antibody (Molecular Probes). Tissues were washed 5 times with PBS and mounted with mounting media containing DAPI (Vectashield®). Alpha smooth muscle actin was detected using the monoclonal antibody clone 1A4 (DAKO). For cell cultures, HepG2 and HLE cells were seeded onto glass coverslips at 3×10^4^ in 24-well plates overnight and treated with LY2157299 or D10 in serum-free medium. Cells were then stimulated with TGF-β1 in the presence or absence of LY2157299 or D10. LY2157299, D10 or TGF-β1 were added daily and after 72 hours cells were fixed with 4% paraformaldehyde in PBS for 10 minutes at room temperature (R.T.) and live-imaged under a phase contrast microscope (Olympus). For immunofluorescence, cells were fixed, washed with PBS, permeabilized with 0.2% Triton X-100 and stained with both TRITC-phalloidin and anti-E-cadherin antibody (BD Transduction Laboratories™). Stained cells were examined under a NIKON eclipse E1000 fluorescence microscope.

### Statistical Analysis

Statistical analyses of the migration, invasion and mRNA were performed using two-tailed Student t test or ANOVA (analysis of variance) and statistical significance was set at a P value of <0.05. All experimental data are described as the mean ±SD. Qualitative variables are summarized as counts and percentages. Associations between variables were assessed using the chi-square test or two-sided Fisher's exact test as appropriate. Results were considered statistically significant at p-values <0.05. Analyses were performed with SAS 9.3 software for Windows OS personal computer.

## Results

**Figure 1:**
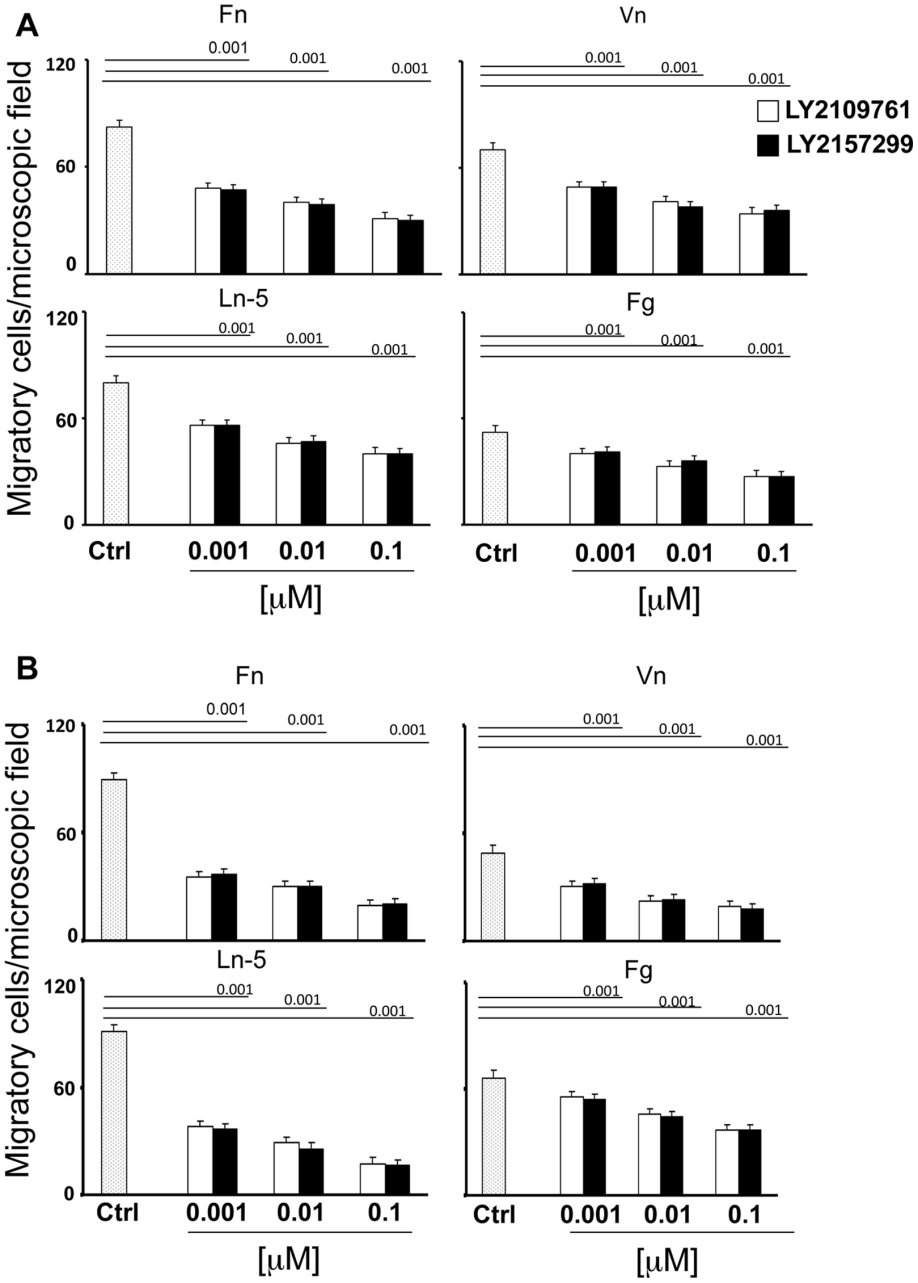
Comparison of the effect of two TGF-βR inhibitor analogs, LY2109761 and LY2157299, on HCC cell migration on different extracellular matrix components. (A) HLE and (B) HLF cells were allowed to migrate for 16 hours on fibronectin (Fn), vitronectin (Vn), laminin-5 (Ln-5) and fibrinogen (Fg) in the presence or absence (vehicle) of increasing concentrations of LY2109761 or LY2157299. All conditions were performed in duplicate, and each experiment was repeated at least three times. Results of representative experiments are shown, and values are expressed as mean ± standard deviation.

**Figure 2:**
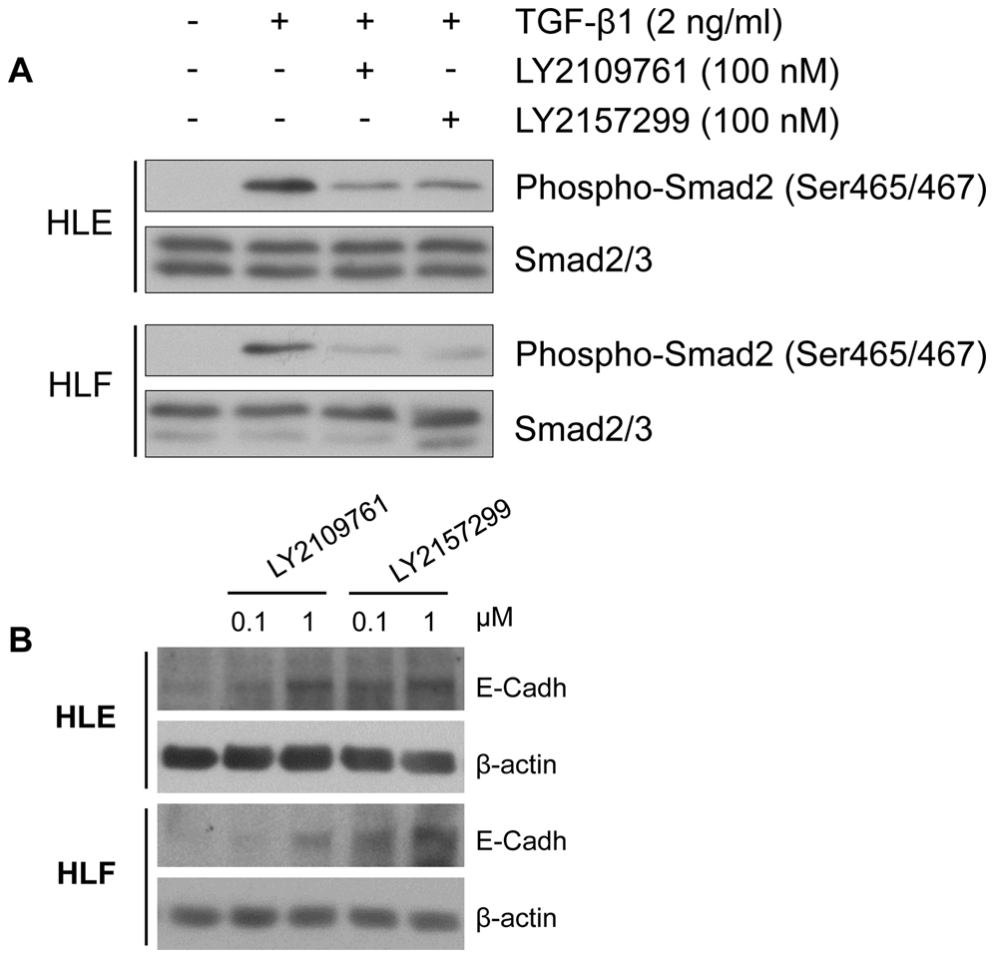
Effect of LY2109761 and LY2157299 on SMAD2 phosphorylation upon TGF-β1. (A) Two different HCC cell lines HLE and HLF were pretreated for 16 hours with 100 nM of LY2109761 or LY2157299 and then stimulated with 2 ng of TGF-β1 for 30 min. Phosphorylation of SMAD2 was detected in immunoblots using a rabbit polyclonal antibody directed against Phospho-Smad2 (Ser465/467). (B) Effect of LY2109761 and LY2157299 on E-cadherin expression in HLE and HLF. Increasing expression of E-cadherin is observed in HLE and HLF cells after treatment with both inhibitors for 48 hours.

After having observed that LY2109761 had an effect of reducing HCC tumor progression [10], we tested LY2157299, a novel and second TGF-βRI kinase inhibitor. LY2157299 is a structural analog of LY2109761 with increased water solubility. Firstly, we allowed HLE and HLF cells to migrate on different extracellular matrix (ECM) components such as Fn, Vn, Ln-5 and Fg, in the presence of LY2109761 or LY2157299 at increasing concentrations. All the experimental conditions were identical for treatments with both compounds. Like LY2109761, LY2157299 significantly inhibited (p<0.001) HLE and HLF migration on different ECM substrates (Fig. 1) [12]. We found that the HCC migration inhibiting effect was dose-dependent for both drugs. In addition, LY2157299 and LY2109761 inhibited de novo phosphorylation of p-SMAD2 at the same efficiency in HLE and HLF after stimulation with TGF-β1 (Fig. 2A). Further, both LY2157299 and LY2109761 increased the expression of E-cadherin in both HCC cell lines in a dose-dependent manner (Fig. 2B). Taken together, these data demonstrate that both LY2157299 and LY2109761 have similar functional and biochemical effects. Because of its improved solubility, LY2157299 may be a more suitable candidate for TGF-β inhibition in vivo, including use in clinical trials in humans.

**Figure 3:**
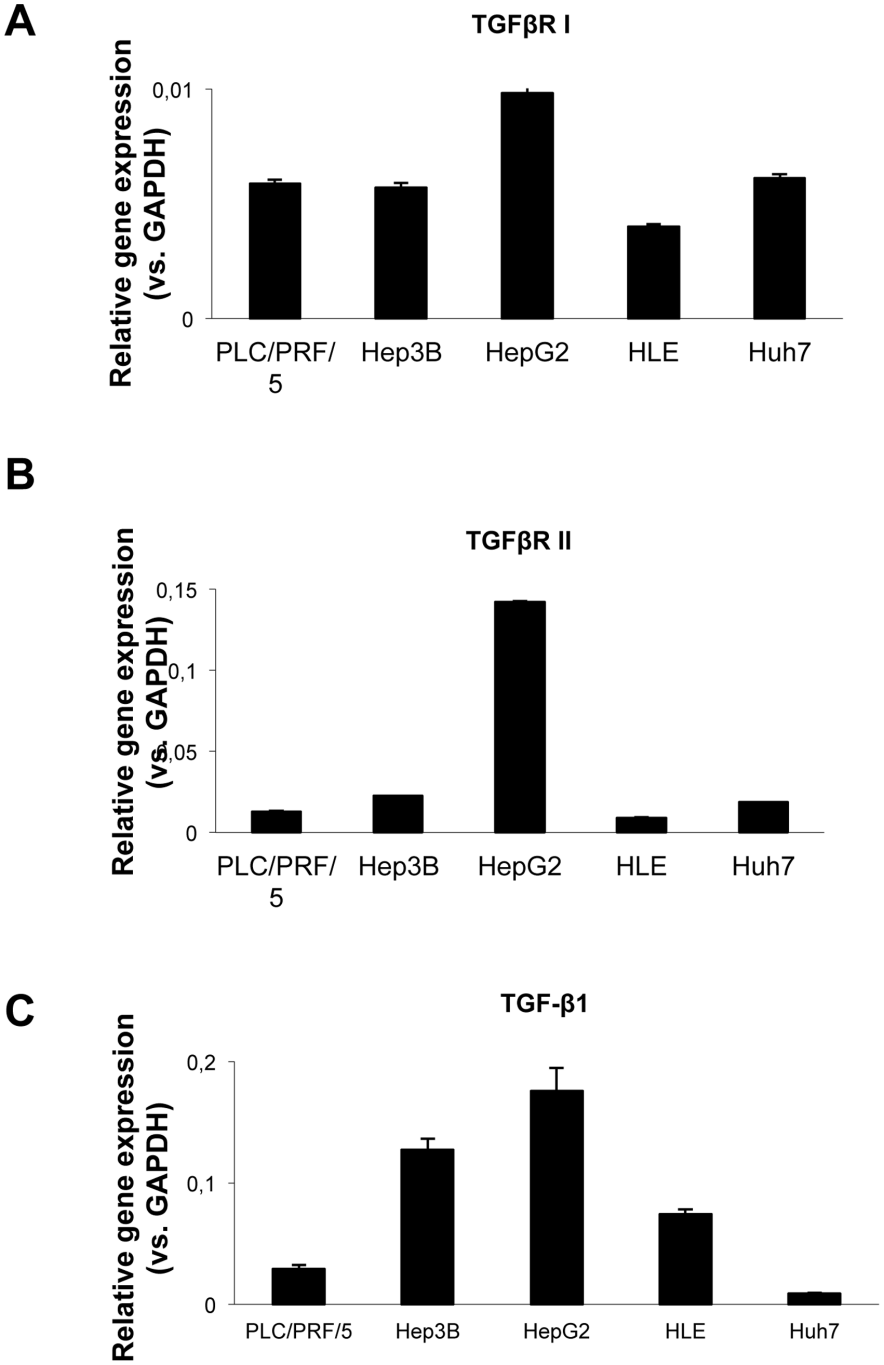
RNA expression levels of TGF-β receptor I, TGF-β receptor II and TGF-β1 in different HCC cell lines evaluated by real-time PCR. Values are expressed as relative gene expression versus GAPDH and represent the mean ±SD of three independent experiments.

We next compared the TGF-βRI inhibitor LY2157299 to D10, a monoclonal antibody directed against TGF-βRII. Firstly, we screened five different HCC cell lines for the expression of TGF-βRI, TGF-βRII and TGF-β1 mRNA levels, to define the most appropriate experimental model. HepG2 cells expressed the highest levels of TGF-β receptors, TGF-βRI and TGF-βRII, as well as TGF-β1, as compared to the other cell lines, Fig. 3.

**Figure 4:**
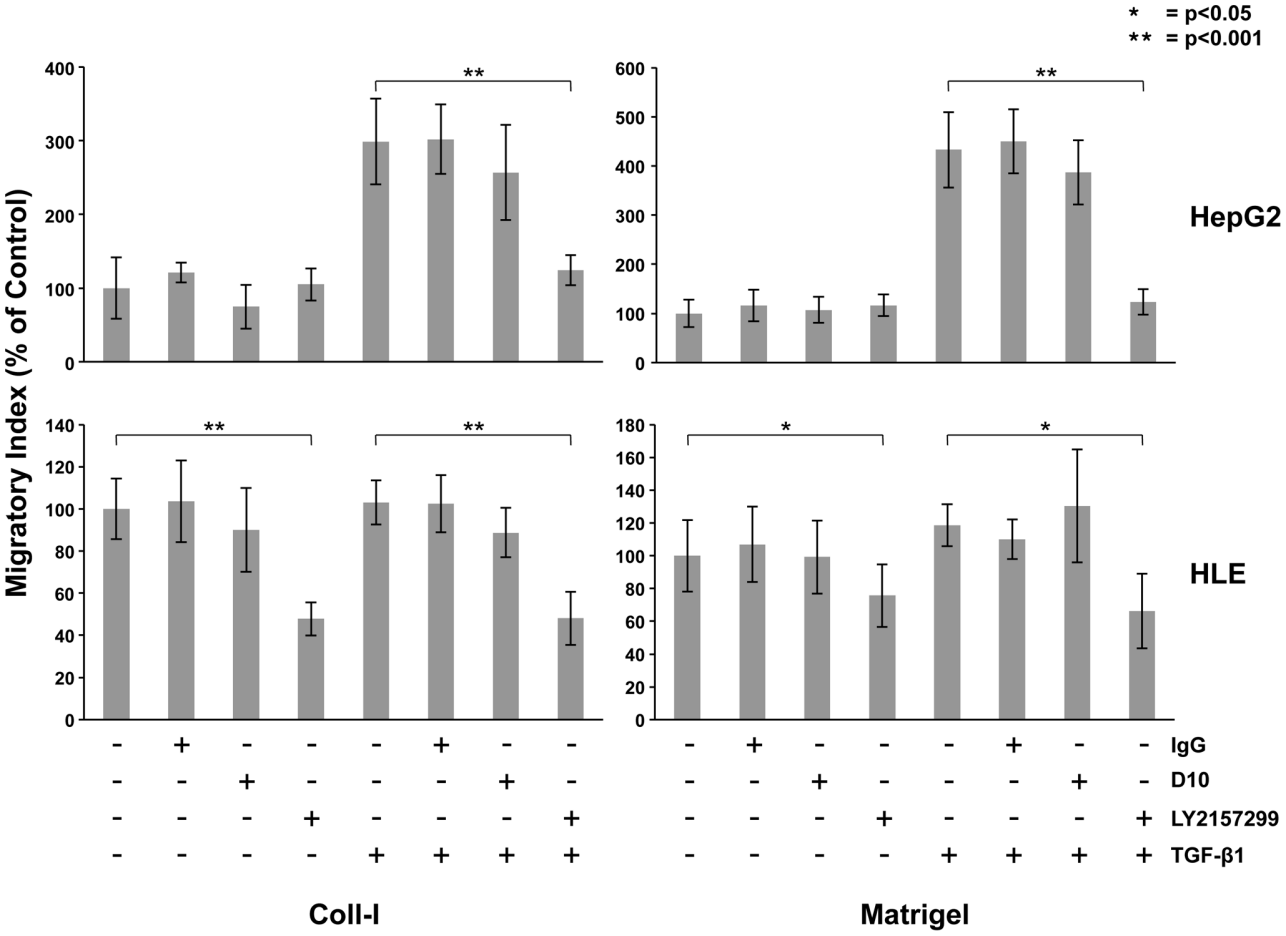
Comparison of the effect between LY2157299 and the fully humanized monoclonal antibody D10 in HCC cell migration. LY2157299 and D10 were tested in two different HCC cell lines, (A) HepG2 and (B) HLE, that were allowed to migrate on collagen I or matrigel. LY2157299, but not D10, was able to inhibit cell migration of both cell lines on both extracellular matrix components. An anti-isotype IgG was used as internal negative control. Results of representative experiments are shown, and values are expressed as mean ± standard deviation. *P<0.05 **P<0.001 versus TGF-β1.

To evaluate the drug effectiveness of LY2157299 and D10, we selected two different HCC cell lines, HepG2 with the highest and HLE with the lowest expression of TGF-βRI and TGF-βRII. HepG2 cells have a constitutively low migratory activity on Coll I and Matrigel. Addition of TGF-β1 significantly increases migration of these cells on Coll I and Matrigel (p<0.001). Surprisingly, treatment with D10 did not antagonize the effect of TGF-β1 on HepG2 migration, whereas LY2157299 efficiently inhibited this effect (p<0.001). HLE cells have a constitutively highly migratory activity on Coll I and Matrigel and are not responsive to the effect of exogenous TGF-β1. However, as in HepG2, we found that LY2157299 efficiently (p<0.001) inhibited migration of HLE, whereas D10 did not. Despite the fact that addition of exogenous TGF-β1 did not increase HLE motility, LY2157299 was still able to inhibit cell migration in this cell line, Fig. 4. Together, these data indicate that LY2157299 is more efficient than D10 in inhibiting cell migration in HCC. On the contrary LY2109761, LY2157299 and D10 did not display any effect on cell proliferation and apoptosis, Fig. S1A–B.

**Figure 5:**
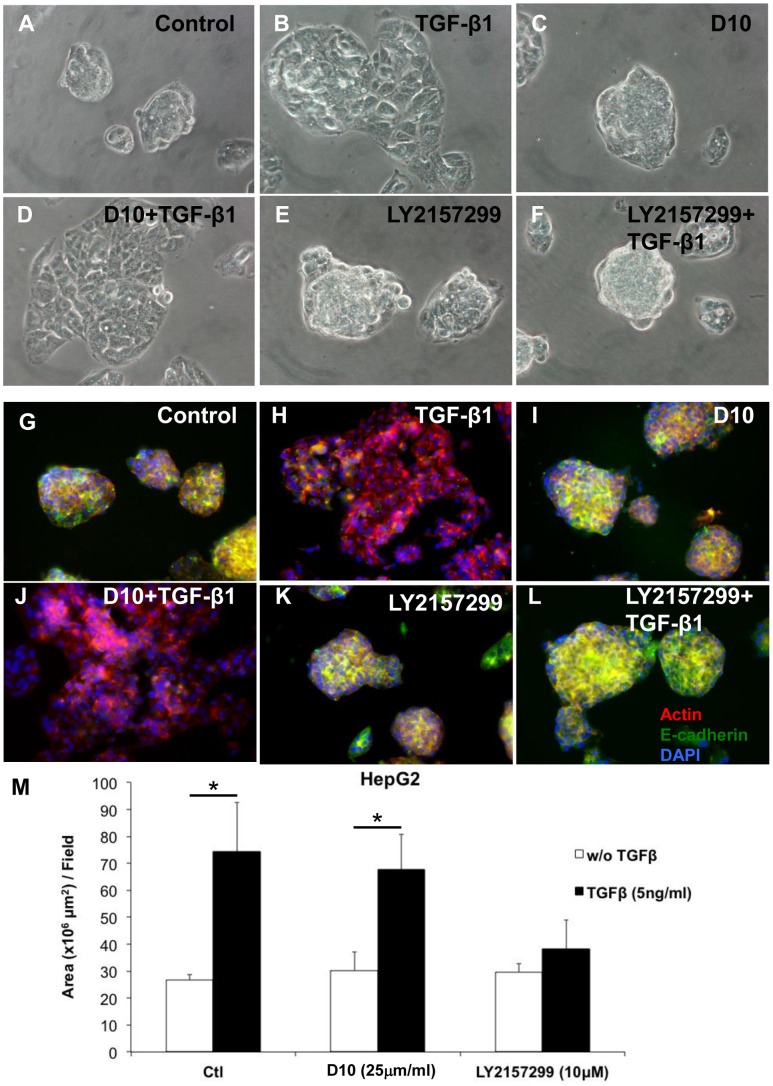
Comparison of the effect between LY2157299 and the fully humanized monoclonal antibody D10 on HCC cell morphology, actin cytoskeleton and E-cadherin expression. HepG2 cells were seeded onto glass coverslips at 3 x104 cells/ml in 24-well plates overnight and treated with control (A, G) or LY2157299 (E, K) or D10 (C, I) alone in serum-free medium. Then, cells were stimulated with TGF-β1 in the absence (B, H) or presence of LY2157299 (F, L) or D10 (D, J). LY2157299, D10 or TGF-β1 were added daily and after 72 hours cells were live-imaged under a phase contrast microscope. For immunofluorescence, cells were fixed with 4% paraformaldehyde, stained with TRITC-phalloidin and anti-E-cadherin antibody and examined under a NIKON eclipse E1000 fluorescence microscope. (M) Cell area was quantified using a combination of two digital image analysis softwares (ImageJ and Adobe Photoshop) and results were plotted as cell area (µm^2^) per random field.**P<0.01.

*In vitro*, HepG2 cells grow in a pile-up anchorage-dependent manner, leading to the formation of three-dimensional nodule-like structures resembling the appearance of cancerous nodules in patients with HCC. In the presence of exogenously added TGF-β1, the architectural structure of these nodule-like masses is remarkably rearranged. HepG2 cells grow in a flat-down manner, and the nodule-like structures become larger. This effect is not reversed by the presence of D10, whereas LY2157299 is able to completely reverse the effect of TGF-β1. As a consequence, HepG2 cells appear similar to their untreated rounded, nodule-like cellular morphology (Fig. 5A–F and Fig. 5M). In the same experimental conditions, HepG2 cells were stained for actin and E-cadherin. In control conditions, E-cadherin is mainly localized on the cellular surface at cell-cell contacts, mainly co-localized with the cortical actin. In the presence of TGF-β1, E-cadherin was no longer detected and actin was localized both at the cell surface and in the cytosol. Addition of D10 to exogenous TGF-β1 did not exert any effect and the cells appeared just like cells treated with TGF-β1 alone. In contrast to D10 treatment, LY2157299 efficiently blocked the effect of exogenous TGF-β1 and E-cadherin was co-localized with cortical actin similarly to controls, Fig. 5G–L.

**Figure 6:**
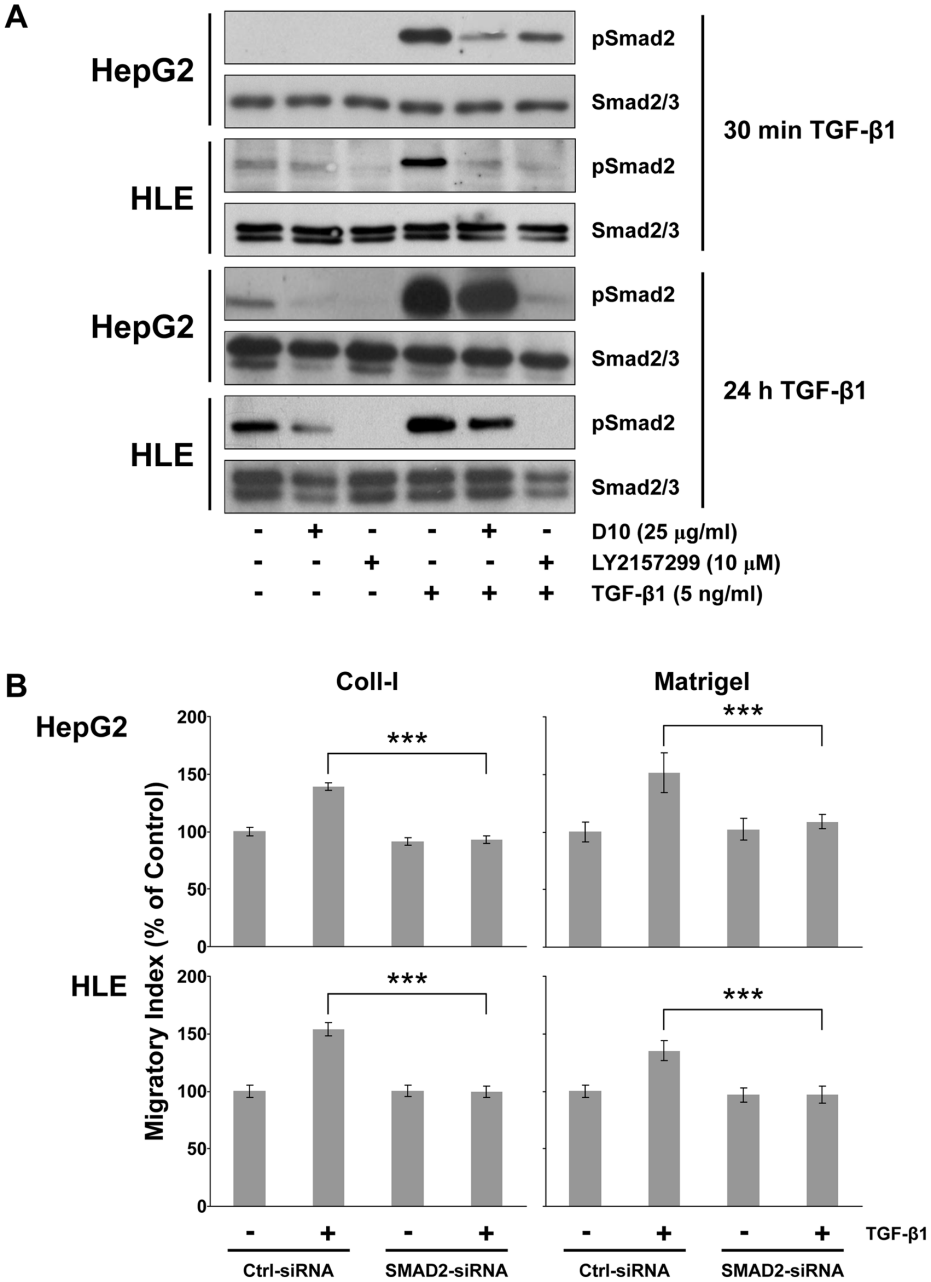
Effect of LY2157299 and D10 on SMAD2 activity in HCC cells. (A) HepG2 and HLE cells were preincubated with LY2157299 (10 µM) or D10 (25 ng/mL) and then stimulated with TGF-β1 (5 ng/mL) in the presence or absence of LY2157299 or D10 for 30 min or 24 hours. Proteins were extracted and western blotting analysis was performed. Phosphorylation of SMAD2 was detected using a rabbit polyclonal antibody directed against phospho-Smad2 (Ser465/467). (B) HepG2 and HLE cells were silenced for SMAD2, or non-silencing control and cell migration assay was performed. Migrated cells were indicated as migratory index versus non TGF-β1-stimulated control. ***P<0.001 versus TGF-β1-stimulated control-siRNA.

To better understand why D10 did not have similar effects to the small molecule inhibitors, we investigated pSMAD2 expression after the activation of TGF-βR signaling. After short-term incubation (30 min), exogenous TGF-β1 phosphorylates SMAD2 in both HepG2 and HLE cells. This effect was inhibited by the presence of D10 and LY2157299. However, after long-term incubation (24 hours) with TGF-β1, phosphorylation of SMAD2 was detectable in the presence of D10 treatment, while cells treated with LY2157299 had no pSMAD2 expression (Fig. 6B). To demonstrate the role of pSMAD2 in TGF-β1 triggered motility, we silenced SMAD2 in HLE and HepG2 cells, Fig. 6A. Then, we challenged them to migrate on Coll I and Matrigel in the absence or presence of exogenous added TGF-β1. After SMAD2 knock-down in HLE and HepG2, TGF-β1 did not increase migration on both substrates as compared to scrambled controls, Fig. 6B. In the same experimental settings, SMAD3 knock-down did not affect HLE and HepG2 TGF-β1-dependent migration, Fig. S2. To further explore the role of pSmad-2, we investigated F-actin and pFAK distribution in silenced Smad-2 and Smad-3 HepG2 and HLE cells after stimulation with TGF-β under the same experimental conditions used for migration experiments.

**Figure 7:**
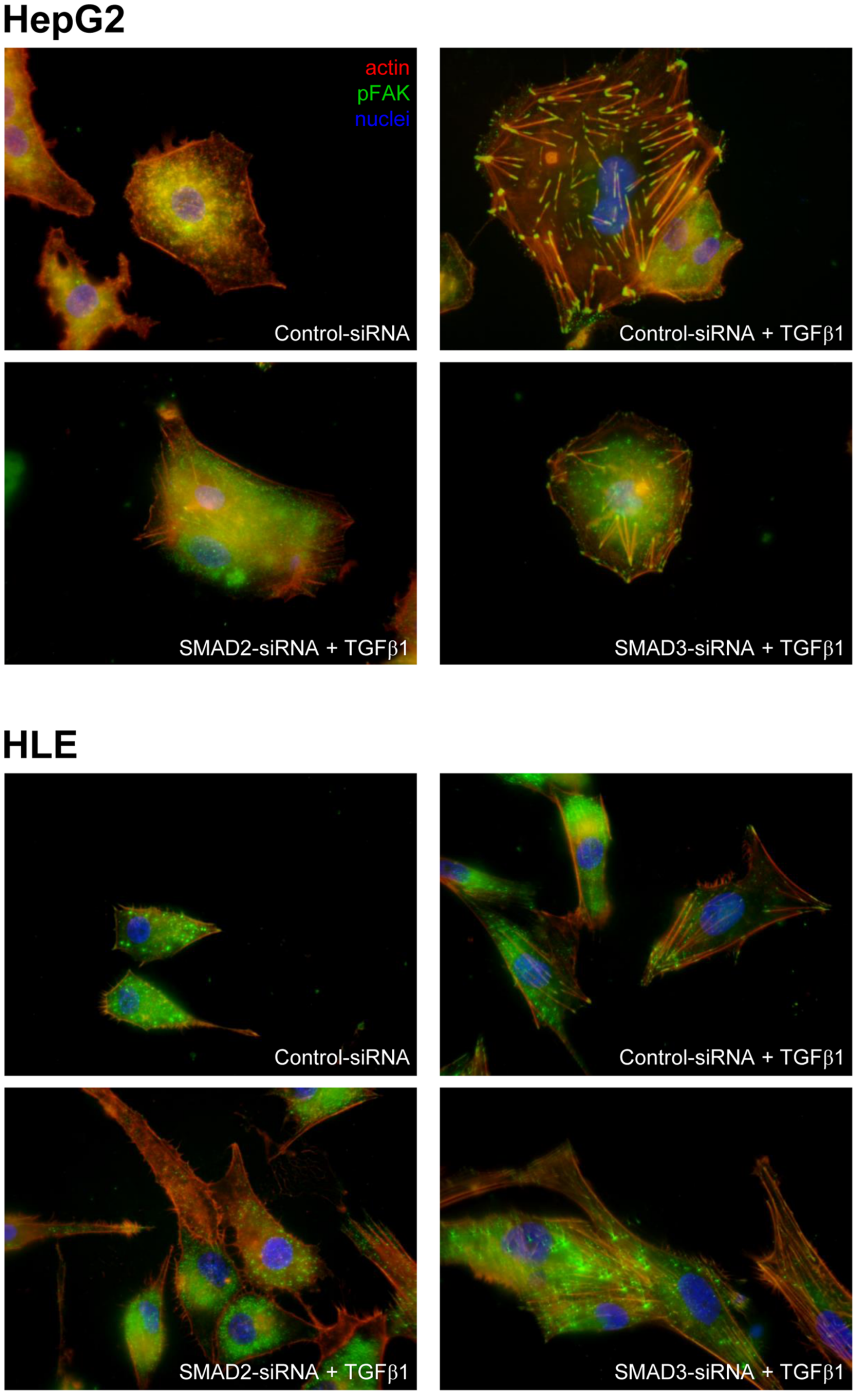
Immunofluorescence staining of F-actin and p-FAK in HepG2 and HLE siRNA for Smad-2 and Smad-3 after stimulation with TGF-β. HepG2 and HLE cells silenced for Smad-2 and Smad-3 were incubated with TGF-β under the same experimental conditions described for migration experiments. Cells were fixed with 4% paraformaldehyde, stained with TRITC-phalloidin and anti-pFAK antibody and examined under a NIKON eclipse E1000 fluorescence microscope. Mag = 600×.

In control siRNA HepG2 and HLE cells, F-actin was distributed mainly cortically along the plasma membrane, while pFAK was scattered throughout the cells. TGF-β treatment altered the cellular ultrastructure, strongly increasing the cytoskeletal stress fibers distributed in the cells, and pFAK formation, clustered at the ends of the stress fibers. The TGF-β mediated effect was completely inhibited by Smad-2 but not by Smad-3 silencing Fig. 7.

**Figure 8:**
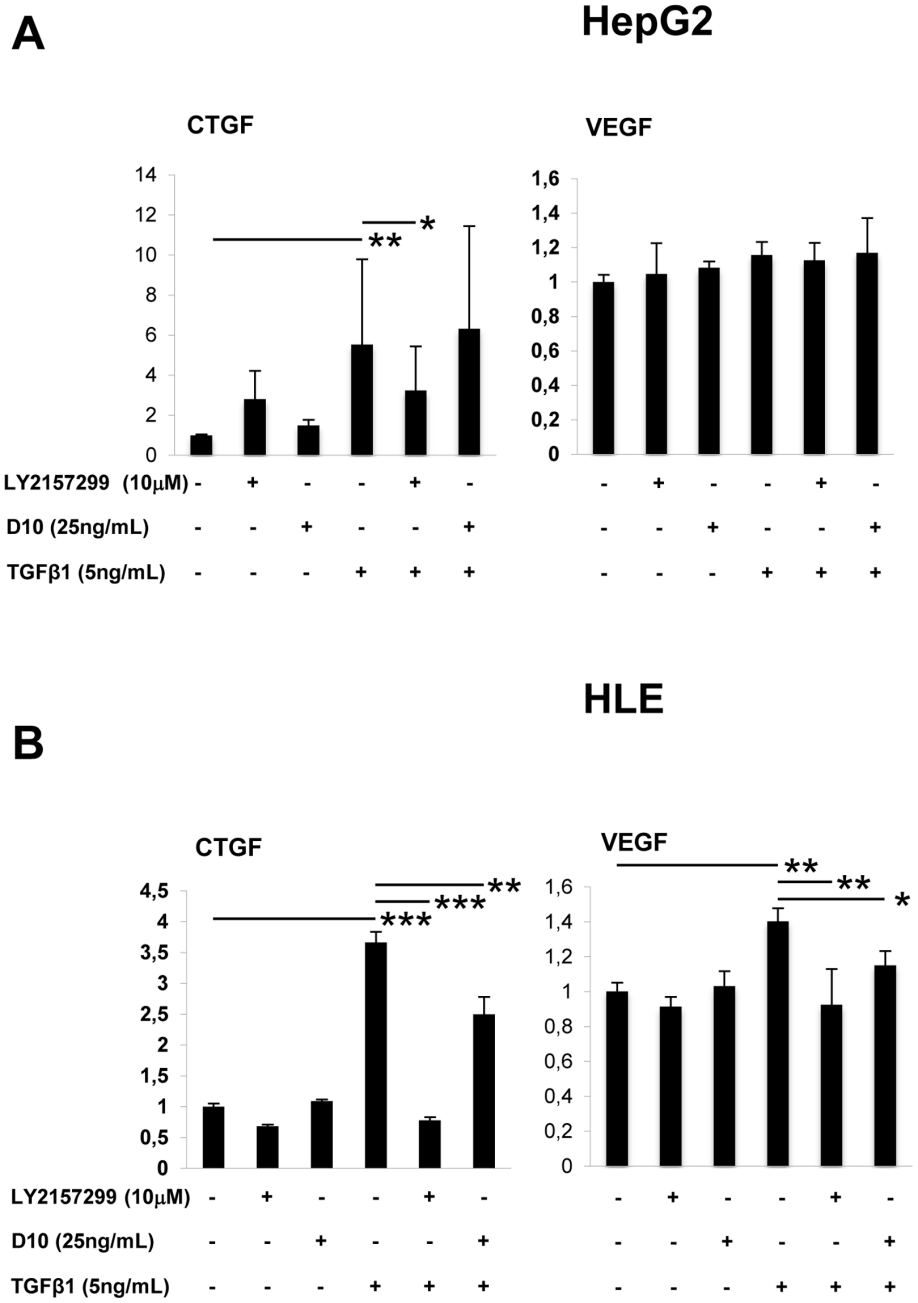
Effect of LY2157299 and D10 on CTGF and VEGF gene expression in HCC cell lines. (A) HepG2 and (B) HLE cells were preincubated with LY2157299 (10 µM) or D10 (25 ng/mL) and then stimulated with TGF-β1 (5 ng/mL) in the presence or absence of LY2157299 or D10. After 48 hours, RNA was extracted and real-time PCR was performed. Values are expressed as relative gene expression versus GAPDH and represent the mean ±SD of three independent experiments. *P<0.05, **P<0.01, ***P<0.001.

These results suggest that pSMAD2 but not pSMAD3 plays a pivotal role in regulating TGF-β1-dependent migration. Furthermore, HCC cells showed up-regulation of MMP-2 mRNA upon TGF-β1 treatment, but knock-down for SMAD2 abolished MMP-2 response, suggesting that MMP-2 is a down stream effector of SMAD2, Fig. S3. Therefore, it is likely that the limited efficacy of D10 as compared to LY2157299 is due to a short-time inhibition of pSMAD2. In addition to these observations, we also compared the effect of LY2157299 and D10 on CTGF and VEGF gene expression of HLE and HepG2 cells. HLE and HepG2 cells treated with TGF-β1 up-regulated CTGF and VEGF mRNA. This effect was blocked by LY2157299, but not by D10 (Fig. 8).

**Figure 9:**
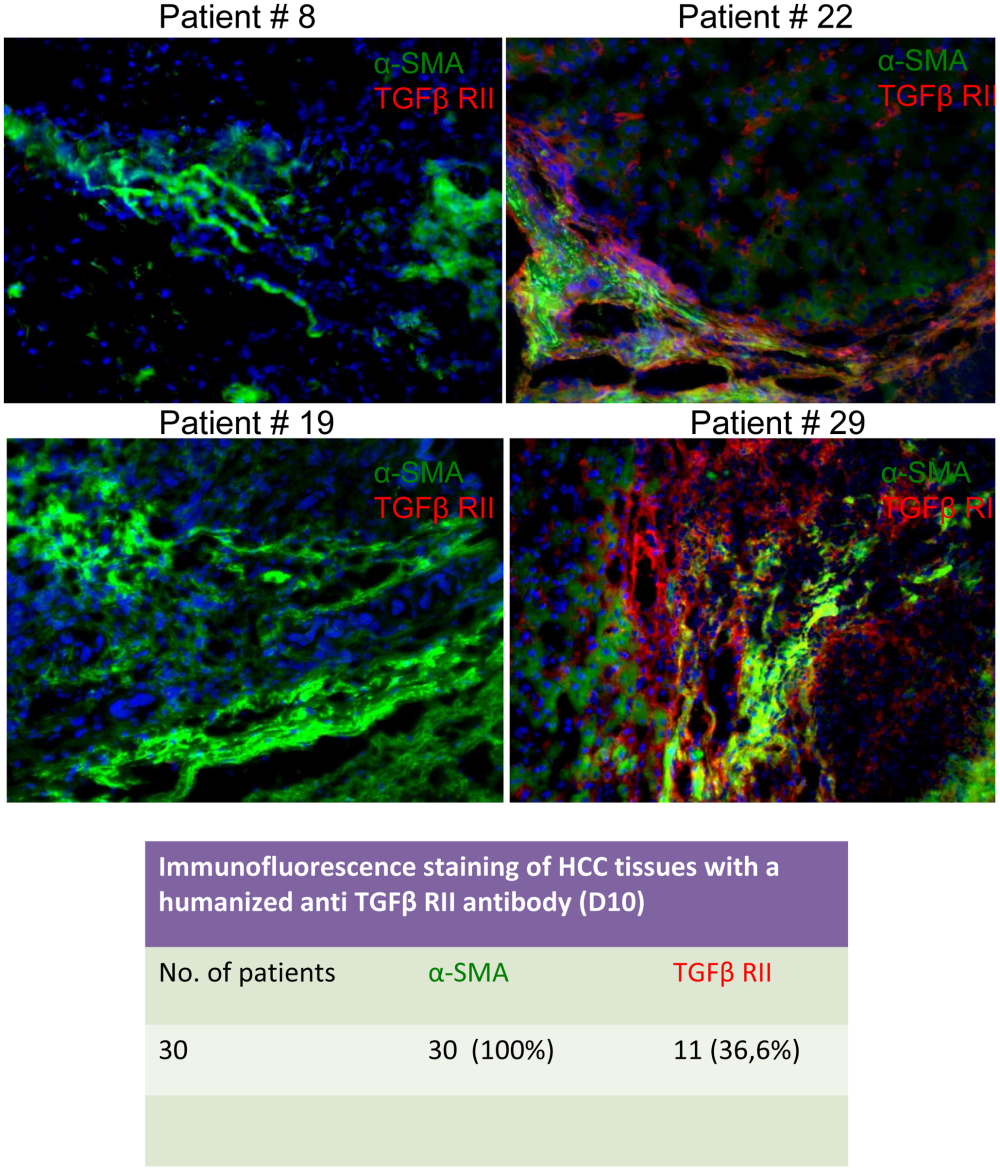
Immunofluorescence staining of TGF-β receptor II in frozen HCC tissues. Tissues from 30 patients with HCC were stained using D10, a fully humanized monoclonal antibody directed against TGF-β receptor II (red) and alpha smooth muscle actin with the monoclonal antibody clone 1A4 (green). In 11 out of 30 HCC tissues (36.6%) positivity for TGF-β receptor II was obtained; representative cases of TGF-β receptor II-positive (patient #22 and #29) or -negative (patients #8 and #19) tissues are shown.

Finally, we investigated the expression of TGF-βRII and p-Smad2 in thirty patients with HCC by immunofluorescence microscopy. To differentiate stromal from cancer cells, we performed double staining with an anti α-SMA antibody, a known marker of myofibroblasts. TGF-βRII was detected with a spotty pattern in the section. It was mainly expressed on the cellular membrane of cancer cells, although it was also occasionally present on myofibroblasts surrounding the tumor, Fig. 9.

**Figure 10:**
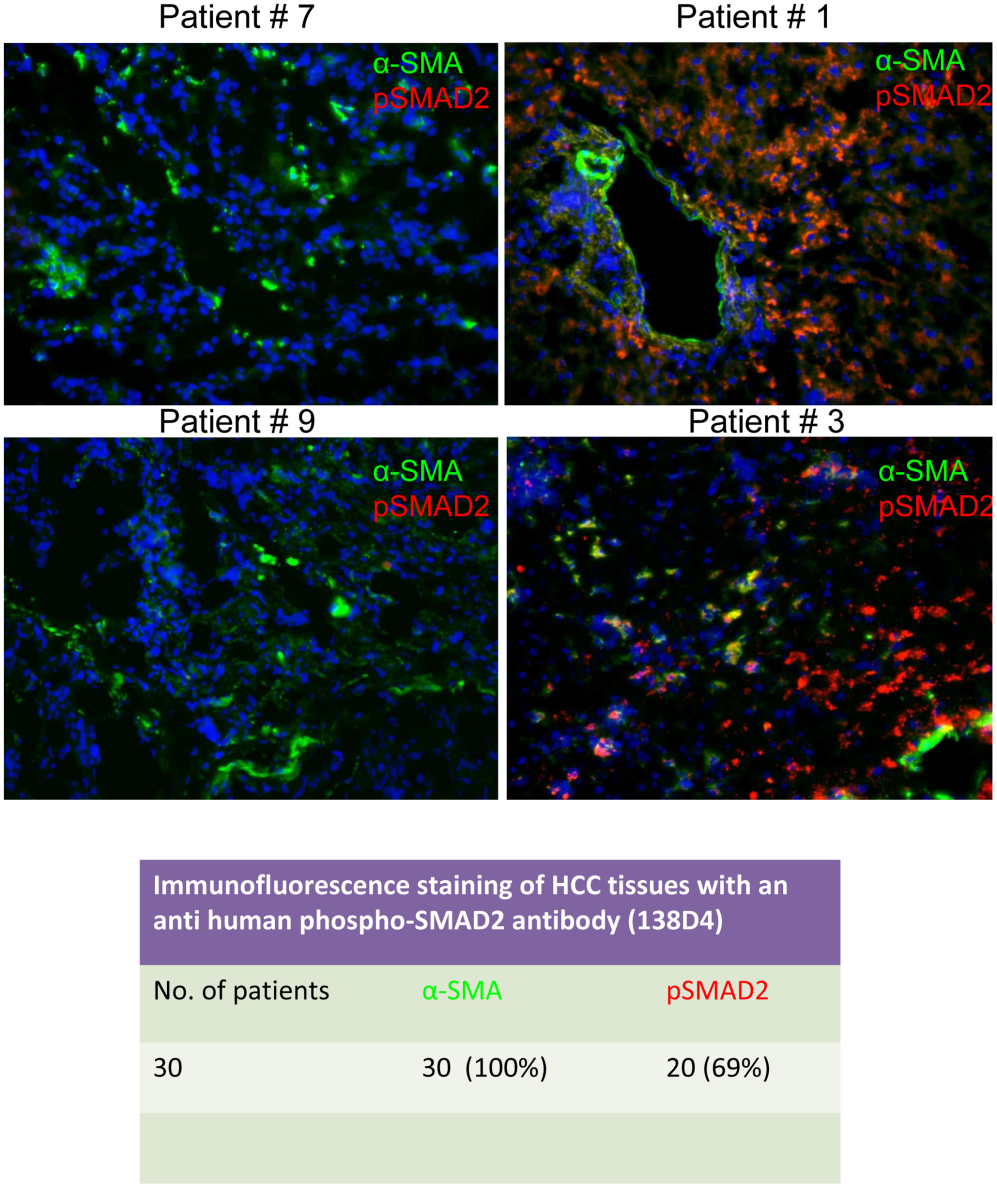
Immunofluorescence staining of p-Smad2 in frozen HCC tissues. Tissues from 30 patients with HCC were stained using an anti-p-Smad2 polyclonal antibody (red) and alpha smooth muscle actin with the monoclonal antibody clone 1A4 (green). In 20 out of 30 HCC tissues (69.0%) positivity for p-Smad2 was obtained; representative cases of p-Smad2-positive (patient #1 and #3) or -negative (patients #7 and #9) tissues are shown.

The staining of p-Smad2 presented a leopard-skin pattern, with strongly positive clusters of cells and other areas with a weak or absent staining. P-Smad2 was detected intracellularly, and limited to cancer cells only, since no co-staining was observed in myofibroblasts for α-SMA and p-Smad2, Fig. 10.

The percentage of patients positive to TGF-βRII was 45.0% (9/20) in HCV positive and 22.2% (2/9) in patients with other etiology; this difference was not statistically significant (Fisher’s exact test: p = 0.412). The percentage of patients positive to TGF-βRII classified as stage A in BCLC was 38.5% (5/13) as compared to 40% in stage B (6/15), again not statistically significant (Fisher’s Exact test: p = 1). There was no statistically significant difference (Fisher’s exact test: p = 1) in the percentage of patients positive to p-Smad2 when comparing HCV positive patients, 70% (14/20) being p-Smad2 positive, with patients having other disease etiology, of which 66.7% (6/9) were pSmad2 positive. The same applied when comparing p-Smad2 among BCLC stages, except C that could not be analysed: 53.8% (7/13) were positive in group A vs 80% (12/15) in group B (Fisher’s exact test: p = 0.227).

The association between TGF-βRII and p-Smad2 resulted barely statistically significant (p = 0.0459): only 1 among the 9 p-Smad2 negative patients resulted positive to TGF-βRII and 10 among the 20 p-Smad2 positive resulted positive to TGF-βRII. Stratified analysis, taking into account the BCLC classification was performed only for stages A and B because only 1 patient resulted BCLC stage C. The association between TGF-βRII and p-Smad2 in BCLC stage A resulted statistically significant (Fisher’s exact test: p = 0.02): among 7 patients positive to p-Smad2, 5 resulted positive to TGF-βRII and among the 6 p-Smad2 negative patients none resulted positive to TGF-βRII. The association between the two biomarkers in BCLC stage B was not statistically significant (Fisher’s exact test: p = 1): among 3 p-Smad2 negative patients only 1 was positive to TGF-β and among 12 p-Smad2 positive patients, 5 resulted positive to TGF-βRII. Table 1.

**Table 1:** Demographic charactersitics of patients.

Patient number	TGF-β RII	p-SMAD 2	Sex	Age (yrs)	Etiology	Child-Pugh	BCLC
1	pos	pos	M	77	HBV	B	A
2	pos	pos	F	69	HCV	A	A
3	pos	pos	M	80	HCV - HBV	A	B
4	neg	pos	M	66	HCV	A	B
5	neg	neg	M	58	HBV	A	B
6	pos	pos	M	69	HCV	A	B
7	pos	pos	F	71	HCV	A	B
8	neg	pos	M	72	Alcol	A	A
9	neg	neg	M	49	HCV	A	B
19	pos	pos	M	53	HCV	A	B
11	neg	pos	F	68	HCV+HBV	B	B
12	neg	pos	M	60	HCV	A	A
13	neg	neg	M	69	HBV	B	A
14	neg	pos	M	41	HBV	B	B
15	neg	neg	F	67	HCV	A	A
16	neg	pos	F	71	HCV	A	B
17	neg	neg	M	65	HCV	B	A
18	pos	pos	M	72	Unknown	B	A
19	neg	neg	M	66	HCV	A	A
20	pos	pos	M	63	HCV	A	A
21	neg	pos	M	73	HBV	A	B
22	neg	pos	M	56	HCV+Alcool	A	B
23	neg	pos	M	63	HCV	A	C
24	pos	neg	M	72	HCV	B	B
25	neg	pos	M	73	Alcool	A	B
26	neg	neg	M	59	Unknown	A	A
27	pos	pos	M	73	HCV	A	B
28	pos	pos	F	67	HCV	B	A
29	neg	neg	M	70	HCV	A	A
30	neg	neg	M	71	HCV	A	A

## Discussion

The possibility of targeting TGF-β signaling in HCC offers a novel and appealing opportunity, because this pathway is implicated in the worsening of the underlying chronic liver disease and the onset of tumor progression [10], [23]. In this study, we show that LY2157299, currently being investigated in a clinical trial phase II (NCT01246986, http://clinicaltrials.gov), acts like LY2109761. We also found several differences between the small molecule inhibitors and the monoclonal antibody targeting TGF-βRII: 1) LY2157299 displays similar functionalities and biochemical activities to those of LY2109761; 2) LY2157299 blocks migration and tumor growth in vitro independently of the expression levels of TGF-βRs, whereas D10 does not; 3) LY2157299 inhibits Smad-2 phosphorylation for a longer time than D10, and pSmad-2 is a key mediator of TGF-β1-triggered motility; 4) in patients with HCC, TGF-βRII is expressed in 33% of the cases.

The effectiveness of LY2157299 and LY2109761 in inhibiting HCC cell migration, and pSMAD2, E-cadherin, VEGF and CTGF dephosphorylation, imply that both molecules share similar activity profiles [13], [20]. This study is the first report demonstrating the effectiveness of LY2157299 on HCC cells *in vitro*, providing a scientific rationale for using this drug in clinical trials. Also, it presents direct comparisons between the two small molecule inhibitors.

Recently, it has been reported that D10, a neutralizing antibody directed against TGF-βRII, blocks breast cancer growth and spread in animal models via inhibition of Smad-2 phosphorylation [21]. Consistently with this report, in our experimental conditions D10 also showed a de-phosphorylating activity on pSMAD2 at short term incubation (30 minutes), but not after a longer time (24 hours), thus explaining its lack of activity on migration measured at similar times.

Interestingly, *in vitro* TGF-β1 rearranges the way that HepG2 cells grow in nodule-like structures, transforming them into larger cellular structures with a consequent down-regulation of E-cadherin expression at cell-cell contact. Also in this case, LY2157299 blocks TGF-β1 effects whereas D10 does not. Because of these *in vitro* changes of HCC nodules, radiographic responses using a modified RECIST [24] may be carefully evaluated and perhaps offer a way to translate this observation into clinical studies with LY2157299.

Finally, another important point is the expression of TGF-βRII. This may help to select patients for treatment with D10. Of 30 patients, we detected the expression of this receptor on tumor parenchyma in one third of the patients. If HCC patients for D10 therapy should be selected based on the expression of TGF-βRII, then a larger study would need to be conducted to confirm that a third of patients express the receptor. In the same group of patients, 69% showed positive staining for p-Smad2, suggesting activation of the pathway. These observations also confirm our in vitro data that activation of TGF-β signaling occurs even if TGF-βRII is apparently not expressed. Our data suggest that the positive staining for p-Smad2 could represent a biomarker for selecting patients more likely to benefit from anti-TGF-β therapy, but further studies are needed to expand and confirm this observation in a larger cohort of patients. At present, these data represent an interesting observation and a preliminary report.

On the other hand it has been reported that TGF-β reduced PLC/PRF/5 cell proliferation in vitro and that a low expression of TGF-βRII in HCC tissues was correlated with a more aggressive disease [11]. This is not entirely surprising considering the well known double-sword effect of TGF-β. Also, the sensitivity of the method should be taken into consideration and the fact that, as we demonstrate in this study, the activation of the signaling can occur following phosphorylation of Smad-2/3 also in the case of low or no expression of TGF-βRII.

Furthermore, it is important to note that the receptor expression on tumor cells is only one aspect in the patients selection approach. The microenvironment and the immune cell expression for this receptor need to be taken into consideration when limiting the selection to tumor tissue expression alone.

In conclusion, we demonstrate that LY2157299 is effective on HCC cells *in vitro*, providing the first evidence to support its use in currently ongoing clinical trials.

## Supporting Information

Figure S1 **Effect of LY2157299 and D10 on proliferation and apoptosis of HCC cell lines.** (A) HepG2 and HLE cells were incubated with LY2109761 (10 µM) or LY2157299 (10 µM) or D10 (25 ng/mL) or IgG1 isotype as negative control (25 ng/mL) and cell proliferation assay was performed on day 6. No effect was observed in the presence of LY2109761 or LY2157299 or D10 compared with control (IgG1 isotype). (B) HepG2 and HLE cells were incubated with LY2109761 (10 µM) or LY2157299 (10 µM) or D10 (25 ng/mL) or IgG1 isotype as negative control (25 ng/mL) and after 4 days of treatment, TUNEL assay was performed. No apoptotic effect was observed in the presence of LY2109761 or LY2157299 or D10 compared with control (IgG isotype). DNAse-treated HLE cells were used as positive control. (C) HepG2 and HLE cells were pre-incubated with an IgG1 isotype for 48 h and then stimulated or not with TGF-β1 (5 ng/mL) for 30 min. Western blot analysis was then performed in the presence or absence of IgG1 isotype or TGF-β1 through collagen I. IgG1 isotype did not affect Smad-2 phosphorylation.
(TIF)

Figure S2 **Silencing of SMAD3 does not affect HCC migration.** (A) SMAD3-knocked-down HepG2 and HLE cells were pre-incubated with TGF-β1 for 48 hours and then allowed to migrate through Collagen-I for 16 hours. (B) Western blot analysis showing silencing of SMAD3 protein in HepG2 and HLE. β-actin was used as loading control. **P<0.01, ***P<0.001.
(TIF)

Figure S3 **MMP-2 is a downstream effector of SMAD2.** MMP-2 mRNA was upregulated in HepG2 cells following TGF-β1 treatment. However, in SMAD2 siRNA cells, treatment with TGF-β1 failed to increase MMP-2 mRNA levels (left panel). SMAD2 silencing was detected by western blotting analysis (right panel). ***P<0.001.
(TIF)

## References

[1] Parkin DM, Bray F, Ferlay J, Pisani P (2005) Global cancer statistics, 2002. CA Cancer J Clin 55: 74–108. 15761078 10.3322/canjclin.55.2.74

[2] de Lope CR, Tremosini S, Forner A, Reig M, Bruix J (2012) Management of HCC. J Hepatol 56 Suppl 1S75–S87 S0168–8278(12)60009-9 [pii];10.1016/S0168-8278(12)60009-9 [doi] 22300468 10.1016/S0168-8278(12)60009-9

[3] Llovet JM, Ricci S, Mazzaferro V, Hilgard P, Gane E, et al (2008) Sorafenib in advanced hepatocellular carcinoma. N Engl J Med 359: 378–390. 18650514 10.1056/NEJMoa0708857

[4] Iavarone M, Cabibbo G, Piscaglia F, Zavaglia C, Grieco A, et al (2011) Field-practice study of sorafenib therapy for hepatocellular carcinoma: a prospective multicenter study in Italy. Hepatology 54: 2055–2063 10.1002/hep.24644 [doi] 21898496 10.1002/hep.24644

[5] Akhurst RJ, Derynck R (2001) TGF-beta signaling in cancer–a double-edged sword. Trends Cell Biol 11: S44–S51. S0962-8924(01)02130-4 [pii]. 10.1016/s0962-8924(01)02130-4 11684442

[6] Colombo M (1999) Natural history and pathogenesis of hepatitis C virus related hepatocellular carcinoma. J Hepatol 31 Suppl 125–30. 10622556 10.1016/s0168-8278(99)80370-5

[7] Bissell MJ, Radisky D (2001) Putting tumours in context. Nat Rev Cancer 1: 46–54. 11900251 10.1038/35094059 PMC2975572

[8] Ito N, Kawata S, Tamura S, Takaishi K, Shirai Y, et al (1991) Elevated levels of transforming growth factor beta messenger RNA and its polypeptide in human hepatocellular carcinoma. Cancer Res 51: 4080–4083. 1649698

[9] Shirai Y, Kawata S, Tamura S, Ito N, Tsushima H, et al (1994) Plasma transforming growth factor-beta 1 in patients with hepatocellular carcinoma. Comparison with chronic liver diseases. Cancer 73: 2275–2279. 7513247 10.1002/1097-0142(19940501)73:9<2275::aid-cncr2820730907>3.0.co;2-t

[10] Giannelli G, Mazzocca A, Fransvea E, Lahn M, Antonaci S (2011) Inhibiting TGF-beta signaling in hepatocellular carcinoma. Biochim Biophys Acta 1815: 214–223 S0304­419X(10)00080-6 [pii];10.1016/j.bbcan.2010.11.004 [doi] 21129443 10.1016/j.bbcan.2010.11.004

[11] Mamiya T, Yamazaki K, Masugi Y, Mori T, Effendi K, et al (2010) Reduced transforming growth factor-beta receptor II expression in hepatocellular carcinoma correlates with intrahepatic metastasis. Lab Invest 90: 1339–1345 labinvest2010105 [pii];10.1038/labinvest.2010.105 [doi] 20531292 10.1038/labinvest.2010.105

[12] Fransvea E, Angelotti U, Antonaci S, Giannelli G (2008) Blocking transforming growth factor-beta up-regulates E-cadherin and reduces migration and invasion of hepatocellular carcinoma cells. Hepatology 47: 1557–1566. 18318443 10.1002/hep.22201

[13] Fransvea E, Mazzocca A, Antonaci S, Giannelli G (2009) Targeting transforming growth factor (TGF)-betaRI inhibits activation of beta1 integrin and blocks vascular invasion in hepatocellular carcinoma. Hepatology 49: 839–850. 19115199 10.1002/hep.22731

[14] Jonas S, Bechstein WO, Steinmuller T, Herrmann M, Radke C, et al (2001) Vascular invasion and histopathologic grading determine outcome after liver transplantation for hepatocellular carcinoma in cirrhosis. Hepatology 33: 1080–1086. 11343235 10.1053/jhep.2001.23561

[15] Giannelli G, Fransvea E, Marinosci F, Bergamini C, Colucci S, et al (2002) Transforming growth factor-beta1 triggers hepatocellular carcinoma invasiveness via alpha3beta1 integrin. Am J Pathol 161: 183–193. 12107103 10.1016/s0002-9440(10)64170-3 PMC1850694

[16] Sumie S, Kuromatsu R, Okuda K, Ando E, Takata A, et al (2008) Microvascular invasion in patients with hepatocellular carcinoma and its predictable clinicopathological factors. Ann Surg Oncol 15: 1375–1382. 18324443 10.1245/s10434-008-9846-9

[17] Mazzocca A, Fransvea E, Lavezzari G, Antonaci S, Giannelli G (2009) Inhibition of transforming growth factor beta receptor I kinase blocks hepatocellular carcinoma growth through neo-angiogenesis regulation. Hepatology 50: 1140–1151. 19711426 10.1002/hep.23118

[18] Abreu JG, Ketpura NI, Reversade B, De Robertis EM (2002) Connective-tissue growth factor (CTGF) modulates cell signalling by BMP and TGF-beta. Nat Cell Biol 4: 599–604. 12134160 10.1038/ncb826 PMC2387275

[19] Kalluri R, Zeisberg M (2006) Fibroblasts in cancer. Nat Rev Cancer 6: 392–401. 16572188 10.1038/nrc1877

[20] Mazzocca A, Fransvea E, Dituri F, Lupo L, Antonaci S, et al (2010) Down-regulation of connective tissue growth factor by inhibition of transforming growth factor beta blocks the tumor-stroma cross-talk and tumor progression in hepatocellular carcinoma. Hepatology 51: 523–534. 19821534 10.1002/hep.23285

[21] Zhong Z, Carroll KD, Policarpio D, Osborn C, Gregory M, et al (2010) Anti-transforming growth factor beta receptor II antibody has therapeutic efficacy against primary tumor growth and metastasis through multieffects on cancer, stroma, and immune cells. Clin Cancer Res 16: 1191–1205 1078–0432.CCR-09-1634 [pii];10.1158/1078-0432.CCR-09-1634 [doi] 20145179 10.1158/1078-0432.CCR-09-1634

[22] Bergamini C, Sgarra C, Trerotoli P, Lupo L, Azzariti A, et al (2007) Laminin-5 stimulates hepatocellular carcinoma growth through a different function of alpha6beta4 and alpha3beta1 integrins. Hepatology 46: 1801–1809. 17948258 10.1002/hep.21936

[23] Bissell DM (2001) Chronic liver injury, TGF-beta, and cancer. Exp Mol Med 33: 179–190. 11795478 10.1038/emm.2001.31

[24] Lencioni R, Llovet JM (2010) Modified RECIST (mRECIST) assessment for hepatocellular carcinoma. Semin Liver Dis 30: 52–60 10.1055/s-0030-1247132 [doi] 20175033 10.1055/s-0030-1247132 PMC12268942

