# Correction: Differential Inhibition of the TGF-β Signaling Pathway in HCC Cells Using the Small Molecule Inhibitor LY2157299 and the D10 Monoclonal Antibody against TGF-β Receptor Type II

**DOI:** 10.1371/annotation/c943a596-3965-4a5b-a27c-55c16685ea32

**Published:** 2013-07-30

**Authors:** Francesco Dituri, Antonio Mazzocca, Joan Fernando, Patrizia Papappicco, Isabel Fabregat, Flavia De Santis, Angelo Paradiso, Carlo Sabbà, Gianluigi Giannelli

The name of the third author was given incorrectly. The correct name is: Joan Fernando. 

The correct citation is: Dituri F, Mazzocca A, Fernando J, Papappicco P, Fabregat I, et al. (2013) Differential Inhibition of the TGF-β Signaling Pathway in HCC Cells Using the Small Molecule Inhibitor LY2157299 and the D10 Monoclonal Antibody against TGF-β Receptor Type II. PLoS ONE 8(6): e67109. doi:10.1371/journal.pone.0067109. 

The correct abbreviation in Contributions is: JF. 

