# Correction: Differential Inhibition of the TGF-β Signaling Pathway in HCC Cells Using the Small Molecule Inhibitor LY2157299 and the D10 Monoclonal Antibody against TGF-β Receptor Type II

**DOI:** 10.1371/journal.pone.0100604

**Published:** 2014-06-12

**Authors:** The PLOS ONE Staff

The authors would like to provide a clarification in relation to Figure 6A in the article.

**Figure 6:**
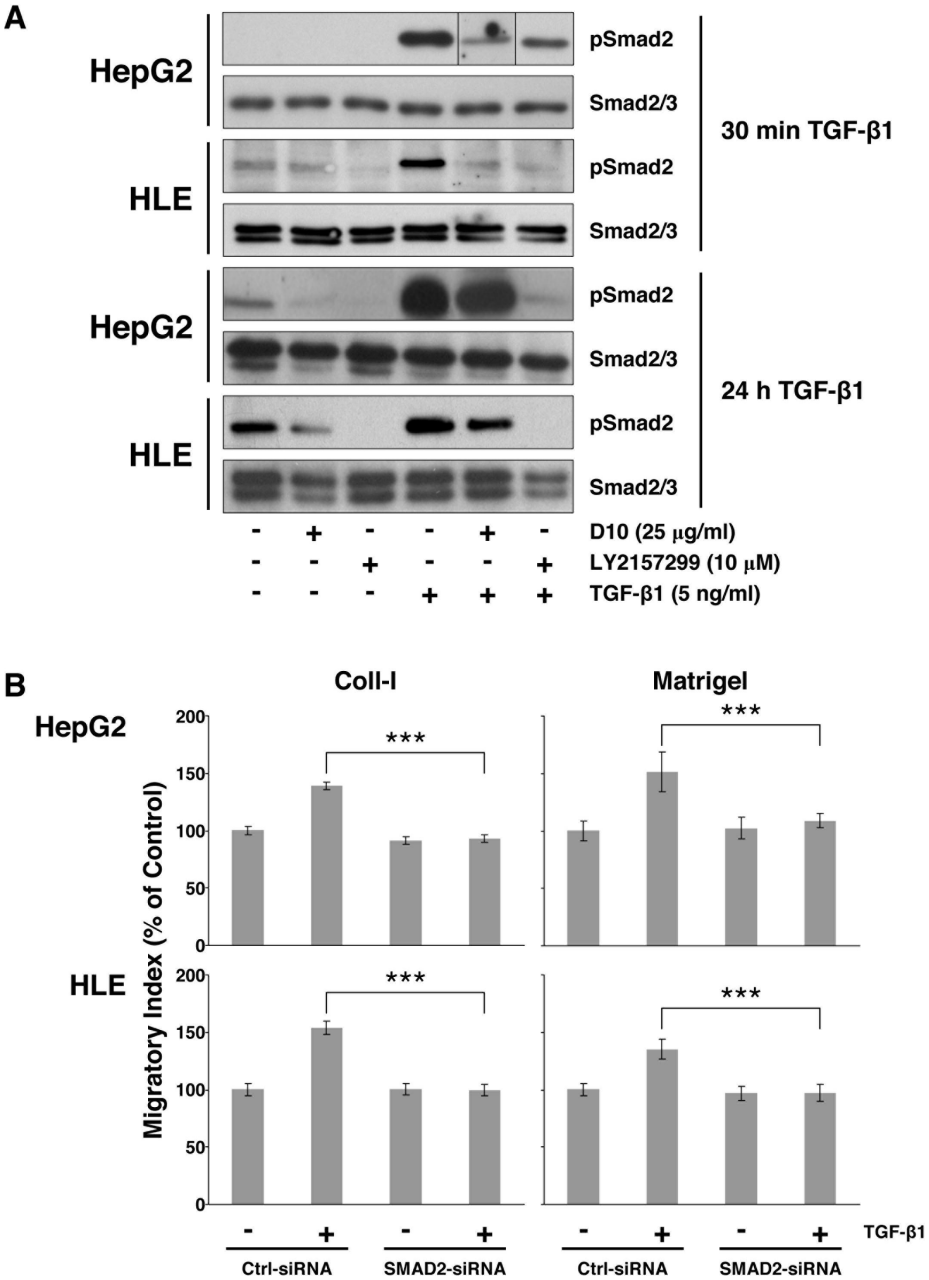
Effect of LY2157299 and D10 on SMAD2 activity in HCC cells. (A) HepG2 and HLE cells were preincubated with LY2157299 (10 µM) or D10 (25 ng/mL) and then stimulated with TGF-β1 (5 ng/mL) in the presence or absence of LY2157299 or D10 for 30 min or 24 hours. Proteins were extracted and western blotting analysis was performed. Phosphorylation of SMAD2 was detected using a rabbit polyclonal antibody directed against phospho-Smad2 (Ser465/467). (B) HepG2 and HLE cells were silenced for SMAD2, or non-silencing control and cell migration assay was performed. Migrated cells were indicated as migratory index versus non TGF-β1-stimulated control. ***P<0.001 versus TGF-β1-stimulated control-siRNA.

The 30 min HepG2 pSmad2 panel in the published article includes a lane (TGFbeta1+D10) that was slightly edited to remove a background spot. In addition, this panel was generated by juxtaposing lanes that were not adjacent in the original blot, this should have been denoted by vertical black lines and the authors apologize for this omission.

The authors are providing a revised version of Figure 6 that denotes the non-adjacent lanes and where the TGFbeta1+D10 lane reflects that in the original blot. The raw blots for the 30 min HepG2 pSmad2 panel are also available via this Correction.

## Supporting Information

Figure S1 Original raw blot for the 30 min HepG2, TGF-beta1, pSmad2 panel (top panel in Figure 6A).

Figure S2 Original raw blot for the 30 min HepG2, TGF-beta1, Smad2/3 panel (second panel in Figure 6A).
