# Supplementary figures and images for: Differential Inhibition of the TGF-β Signaling Pathway in HCC Cells Using the Small Molecule Inhibitor LY2157299 and the D10 Monoclonal Antibody against TGF-β Receptor Type II
Source: PLoS One. 2013 Jun 27;8(6):e67109. doi: 10.1371/journal.pone.0067109 (PMC3694933; doi:10.1371/journal.pone.0067109)

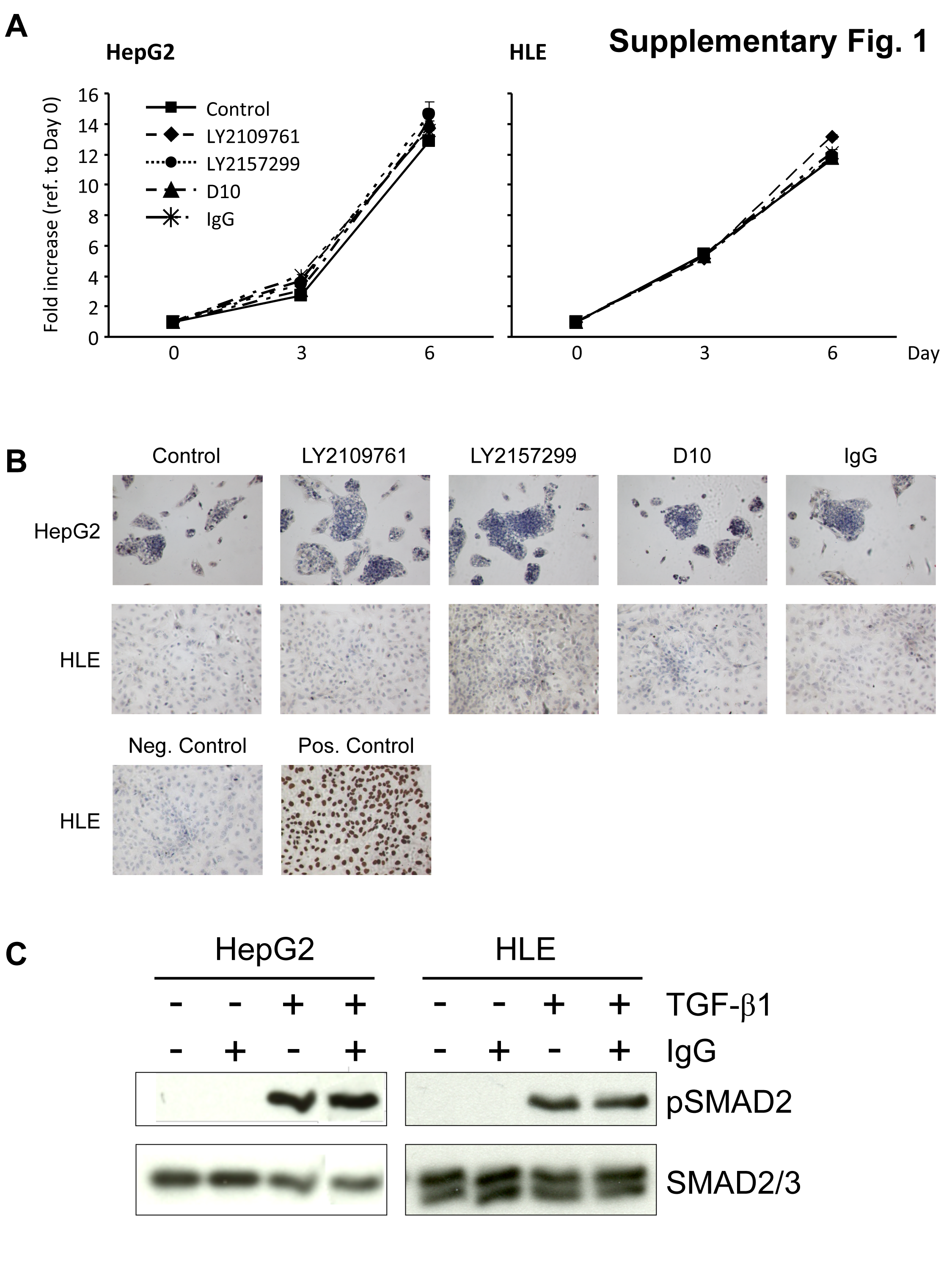

Supplement: Figure S1 — Effect of LY2157299 and D10 on proliferation and apoptosis of HCC cell lines. (A) HepG2 and HLE cells were incubated with LY2109761 (10 µM) or LY2157299 (10 µM) or D10 (25 ng/mL) or IgG1 isotype as negative control (25 ng/mL) and cell proliferation assay was performed on day 6. No effect was observed in the presence of LY2109761 or LY2157299 or D10 compared with control (IgG1 isotype). (B) HepG2 and HLE cells were incubated with LY2109761 (10 µM) or LY2157299 (10 µM) or D10 (25 ng/mL) or IgG1 isotype as negative control (25 ng/mL) and after 4 days of treatment, TUNEL assay was performed. No apoptotic effect was observed in the presence of LY2109761 or LY2157299 or D10 compared with control (IgG isotype). DNAse-treated HLE cells were used as positive control. (C) HepG2 and HLE cells were pre-incubated with an IgG1 isotype for 48 h and then stimulated or not with TGF-β1 (5 ng/mL) for 30 min. Western blot analysis was then performed in the presence or absence of IgG1 isotype or TGF-β1 through collagen I. IgG1 isotype did not affect Smad-2 phosphorylation. (TIF) [file pone.0067109.s001.tif]

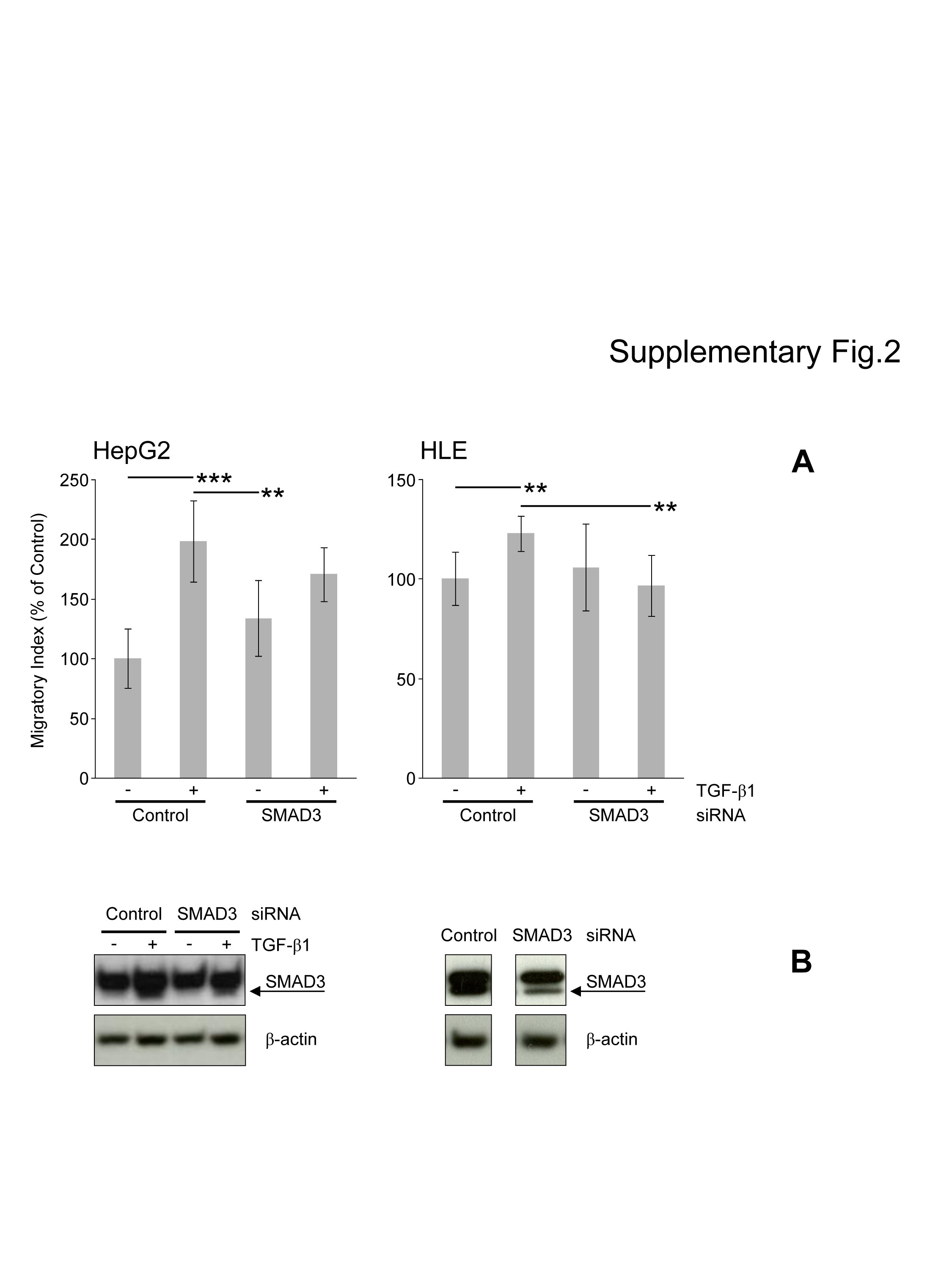

Supplement: Figure S2 — Silencing of SMAD3 does not affect HCC migration. (A) SMAD3-knocked-down HepG2 and HLE cells were pre-incubated with TGF-β1 for 48 hours and then allowed to migrate through Collagen-I for 16 hours. (B) Western blot analysis showing silencing of SMAD3 protein in HepG2 and HLE. β-actin was used as loading control. **P<0.01, ***P<0.001. (TIF) [file pone.0067109.s002.tif]

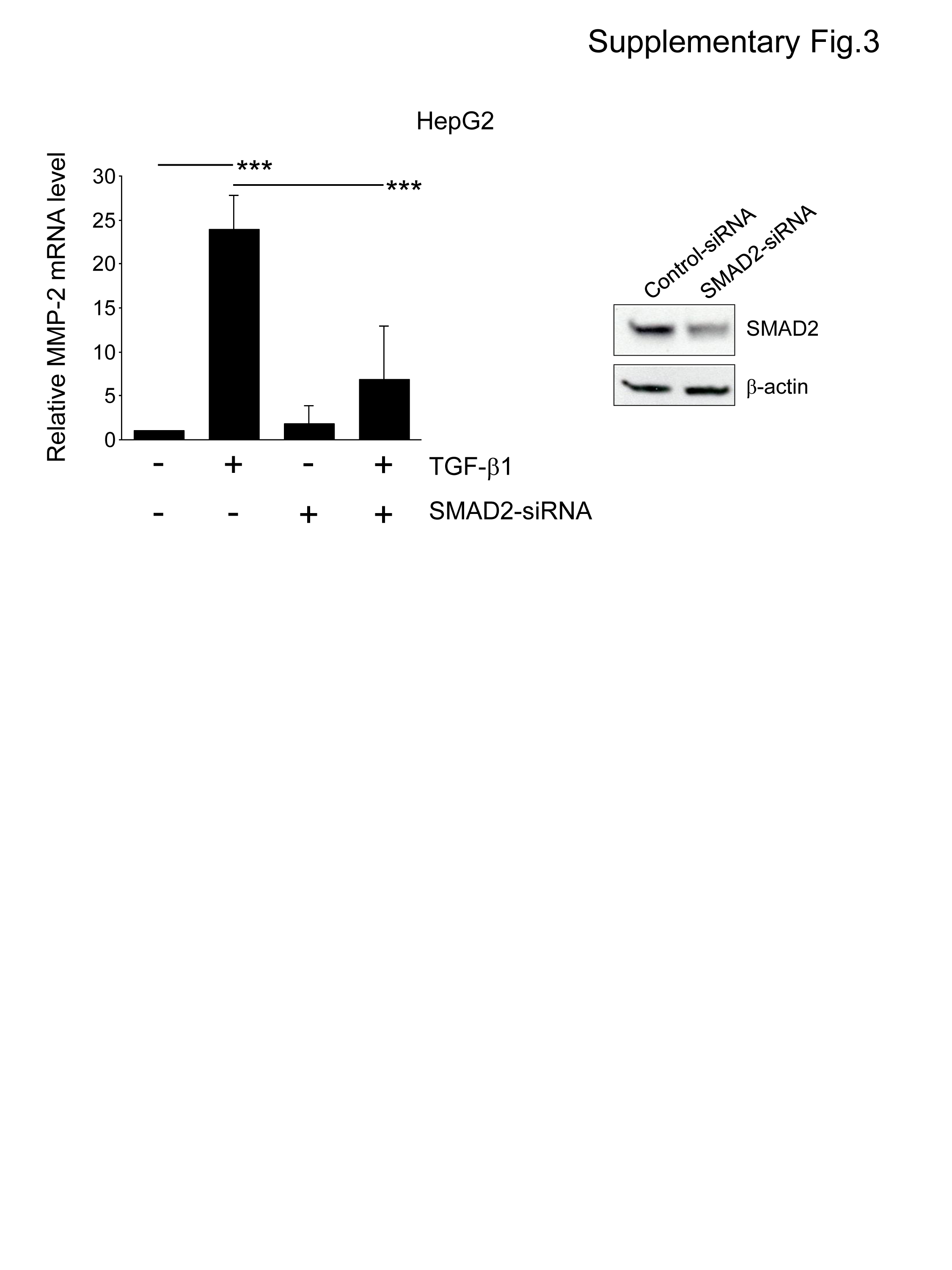

Supplement: Figure S3 — MMP-2 is a downstream effector of SMAD2. MMP-2 mRNA was upregulated in HepG2 cells following TGF-β1 treatment. However, in SMAD2 siRNA cells, treatment with TGF-β1 failed to increase MMP-2 mRNA levels (left panel). SMAD2 silencing was detected by western blotting analysis (right panel). ***P<0.001. (TIF) [file pone.0067109.s003.tif]
