# Supplementary figures and images for: Correction: Differential Inhibition of the TGF-β Signaling Pathway in HCC Cells Using the Small Molecule Inhibitor LY2157299 and the D10 Monoclonal Antibody against TGF-β Receptor Type II
Source: PLoS One. 2014 Jun 12;9(6):e100604. doi: 10.1371/journal.pone.0100604 (PMC4055765; doi:10.1371/journal.pone.0100604)

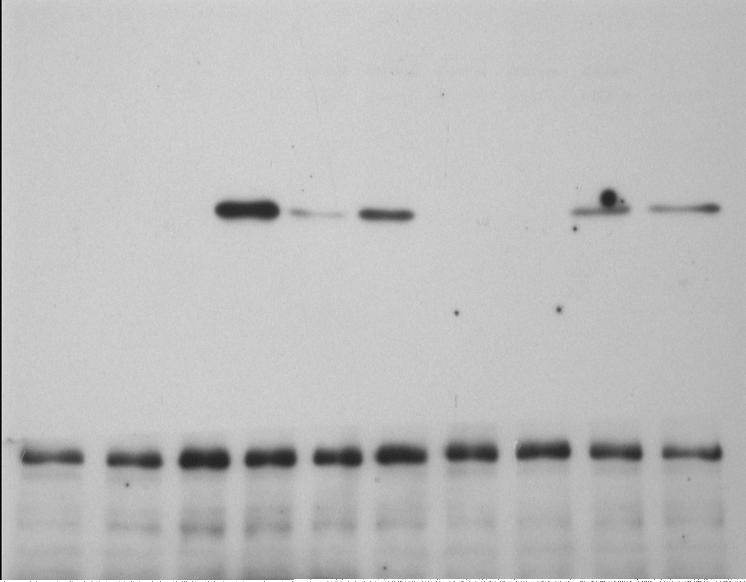

Supplement: Figure S1 — Original raw blot for the 30 min HepG2, TGF-beta1, pSmad2 panel (top panel in Figure 6A). [file pone.0100604.s001.tif]

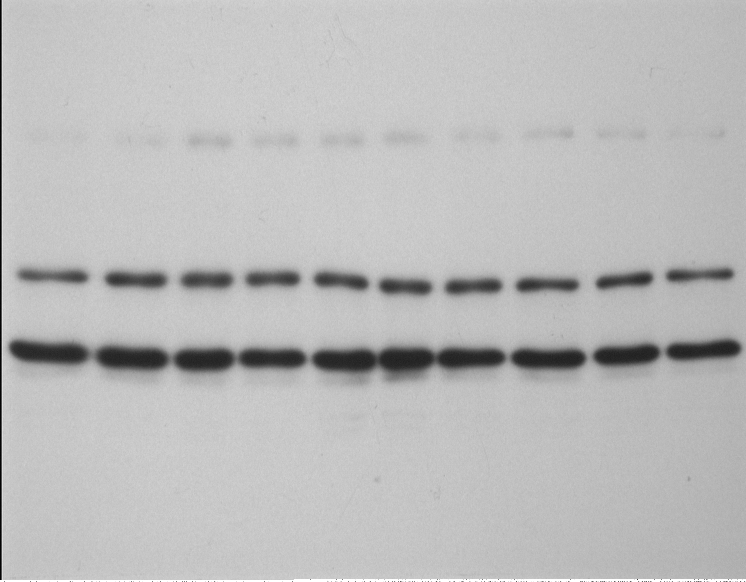

Supplement: Figure S2 — Original raw blot for the 30 min HepG2, TGF-beta1, Smad2/3 panel (second panel in Figure 6A). [file pone.0100604.s002.tif]
